# Targeted Anti-TF Antibody–Drug Conjugates in Advanced Cervical Cancer—Tisotumab Vedotin—Systematic Review

**DOI:** 10.3390/cancers18162685

**Published:** 2026-08-19

**Authors:** Natalia Gierulska, Zuzanna Dąbrowska, Nina Jankowska, Julia Piekarz, Natalia Picheta, Magdalena Skórzewska

**Affiliations:** 1 Student Scientific Society, Department of Clinical Oncology and Chemotherapy, Medical University of Lublin, 20-059 Lublin, Poland; 61669@umlub.edu.pl (N.G.); 61934@umlub.edu.pl (Z.D.); 61976@umlub.edu.pl (N.J.); 59750@umlub.edu.pl (N.P.); 2 Department of Clinical Oncology and Chemotherapy, Medical University of Lublin, 20-059 Lublin, Poland

**Keywords:** tisotumab vedotin, antibody–drug conjugate, cervical cancer, clinical efficacy, drug safety, innovaTV trials

## Abstract

**Background/Objectives**: Cervical cancer (CC) is currently one of the leading causes of cancer-related mortality worldwide. Patients with advanced, recurrent, or metastatic CC, for whom treatment options are limited, are particularly at risk. Tisotumab vedotin (TV) is a first-in-class antibody–drug conjugate targeting tissue factor (TF), representing a promising therapeutic breakthrough. This systematic review aims to comprehensively evaluate the clinical efficacy, safety profile, and therapeutic potential of TV, both as monotherapy and in combination regimens, in patients with advanced cervical cancer. **Methods**: A systematic literature search was conducted in the PubMed, Scopus, and Embase databases, as well as on the ClinicalTrials.gov website, for prospective clinical trials (Phases I–III) in accordance with the PRISMA 2020 guidelines. The search focused on original articles and studies published between 2019 and 2026. Four key clinical trial programmes (innovaTV 201, 204, 205, and 301), involving 548 patients, were selected. Quality assessment was performed using the Cochrane RoB 2.0 tool and the NIH tool for assessing the quality of “before-and-after” studies. **Results**: Objective response rates (ORR) with TV ranged from 17.8% in monotherapy to 65.8% in combination regimens, confirming its efficacy in treatment. In randomised Phase III trials, the use of TV improved overall survival compared to standard chemotherapy. Adverse effects characteristic of TV are primarily ophthalmic complications, peripheral neuropathy, and hemorrhagic events. These were managed through pre-established prophylactic and monitoring protocols. Combination therapies increased therapeutic efficacy but also raised the incidence of grade ≥ 3 adverse events occurring during treatment. **Conclusions**: TV offers new hope by changing the treatment algorithm for cervical cancer. As a targeted therapy, it provides an effective alternative to conventional chemotherapy in previously treated patients. It also has the potential for use in first-line multi-drug treatment regimens. Strict adherence to safety protocols is essential to minimise toxicity and ensure treatment continuity.

## Simple Summary

Cervical cancer remains one of the leading causes of cancer-related deaths among women worldwide, especially in advanced stages of the disease. In these cases in particular, traditional treatments, such as chemotherapy, often become less effective. This study examines the potential of a new targeted drug called tisotumab vedotin (TV) in patients with recurrent or metastatic cervical cancer. By delivering a potent cell-killing agent directly to cancer cells, this drug helps spare healthy tissues. In this study, we evaluate the drug’s efficacy, safety, and potential when combined with immunotherapy and other standard treatments. By synthesising the results of recent clinical trials, we describe how this innovative therapy can improve survival rates and quality of life for patients with limited treatment options. These findings are crucial for improving clinical practice and guiding future research into more personalised and effective cancer treatment strategies.

## 1. Introduction

Cervical cancer (CC) is currently the fourth-most common malignant tumour among women and the most common gynaecological cancer. Despite ever-improving screening, CC remains the leading cause of cancer deaths in women in as many as 37 countries, and ranks fourth globally among women—just behind breast, lung and colorectal cancer. According to the 2022 GLOBOCAN Global Cancer Statistics, as many as 662,000 new cases of cervical cancer were diagnosed [1]. Recent global epidemiological analyses confirm this substantial and persistent burden, alongside marked disparities in screening and HPV vaccination coverage across regions [2]. Currently, in patients with locally advanced disease, the 5-year disease-free survival rate is approximately 68%, and the 5-year overall survival rate is 74%. In advanced stages and with lymph node involvement, the prognosis for patient survival worsens [3]. According to the current ESGO guidelines from 2023, CC treatment should be personalised and depend on the clinical stage of the disease, risk factors and the patient’s desire to preserve fertility. The basis for treatment eligibility is a combination of clinical examination, pathological assessment and advanced imaging studies, primarily including MRI to assess the local tumour and PET-CT or CT to assess lymph nodes and metastases. In young patients who wish to preserve their fertility, the pelvic lymph nodes must first be assessed, preferably via sentinel node biopsies. If the lymph nodes are involved, the patient is not eligible for fertility-sparing treatment (FST). FST in cases of low-grade cancer, i.e., when the tumour is smaller than 2 cm, involves performing a conisation or simple trachelectomy whilst preserving the uterine body, provided that clear surgical margins are achieved. If the cancer is confined to the cervix and the lymph nodes are negative on imaging, this is classified as early-stage cervical cancer. The recommended treatment strategy, and indeed the standard of care in this case, is radical hysterectomy combined with bilateral pelvic lymphadenectomy (PALND) and sentinel node mapping. It is also crucial in this case to agree on adjuvant radiotherapy or definitive concurrent chemoradiotherapy (CTRT), based on cisplatin combined with brachytherapy, if the histopathological examination reveals risk factors. In cases of tumour invasion into the parametrium, vagina or pelvic walls, or radiologically positive lymph nodes, we are dealing with locally advanced cervical cancer (LACC); we use CTRT or image- guided adaptive brachytherapy (IGABT). Routine hysterectomy following successful CTRT is not recommended due to increased morbidity without any benefit in overall survival (OS). In some patients, CC is incidentally diagnosed during a simple hysterectomy. In such cases, radical excision of the parametrium and upper vagina should be performed, followed by PALND or CTRT. The choice of option will depend on the status of the surgical margins and the histopathological assessment of the tumour. In the case of recurrent or metastatic disease, we must adapt the treatment regimen depending on the previous treatments the patient has undergone. Additionally, we must also assess the patient’s current functional status and the anatomical location of the metastasis or recurrence. If the patient has not previously received radiotherapy, the standard approach is salvage radiotherapy. In contrast, for isolated localised lesions, pelvic exenteration may be performed. If the lesions are widely disseminated, the patient must initially receive targeted therapy or systemic chemotherapy [4]. Unfortunately, patients with recurrent or metastatic CC have a poor prognosis, and their treatment options are very limited due to pre-existing resistance to chemotherapy and renal damage. Furthermore, patients who have undergone radiotherapy may experience tissue and vascular damage, which further complicates the choice of treatment. Currently, immunotherapy and targeted therapy offer new hope for patients [5]. Recent reviews have summarised the growing role of immune checkpoint inhibitors and antibody–drug conjugates across first- and second-line treatment of advanced, recurrent, and persistent disease [6]. As these technologies have developed, a new class of drugs has emerged that delivers cytotoxic agents directly to solid tumours—antibody–drug conjugates (ADCs). ADCs consist of three components: a monoclonal antibody (mAb), which selectively binds to tumour antigens on the surface of cancer cells, a linker, and a cytotoxic payload [7]. The linker covalently binds the payload to the antibody, remaining stable in the bloodstream. This prevents premature release of the drug. Subsequently, the linker is cleaved following internalisation of the conjugate by the target cell, enabling the selective destruction of tumour cells whilst sparing healthy tissues [8,9].

A new drug classified as an ADC is Tisotumab Vetodin (TV). It offers new hope for patients with recurrent and metastatic cervical cancer. TV works by directly targeting the tissue factor (TF) on the surface of cancer cells whilst binding to a potent cytotoxic drug—monomethylauristatin E (MMAE). Based on the results of the Phase III innovaTV 301 trial, which demonstrated a significant improvement in overall survival compared with chemotherapy, the drug received accelerated approval from the FDA in September 2021. It subsequently received full approval from the FDA in April 2024 for adult female patients with recurrent or metastatic cervical cancer whose disease had progressed during or after chemotherapy [10,11,12,13]. In March 2025, the European Commission granted a marketing authorisation for TV for the same indication, making it the first antibody–drug conjugate approved in the European Union for use in recurrent or metastatic cervical cancer [10,11,12,13]. TV is currently approved as monotherapy in this group of previously treated patients. Its use in combination regimens and in earlier lines of treatment is still the subject of clinical trials. Recent studies have shown an overall survival (OS) of 11.83 months and a progression-free survival (PFS) of 4.22 months [13].

In this article, we would like to focus on discussing the drug TV and presenting the latest research findings, which offer great hope to patients suffering from recurrent and metastatic cervical cancer compared to current treatment methods.

## 2. Materials and Methods

### 2.1. Study Designs

The systematic review was conducted in accordance with the Preferred Reporting Items for Systematic Reviews and Meta-Analyses (PRISMA) 2020 guidelines (Supplementary Materials). The aim of the review was to comprehensively assess the clinical efficacy, safety, and immune-modulating potential of TV in CC, particularly emphasising its synergistic role when combined with immunotherapy.

The analysis included prospective clinical trial publications evaluating TV in adult patients with CC, including randomised phase III trials, as well as single-arm or multi- cohort phase I, Ib, and II clinical studies as well as long-term follow-up reports, predefined subgroup analyses, and patient-reported outcome (PRO) analyses derived from the same parent trial programmes.

The review protocol was registered in the International Prospective Register of Systematic Reviews (PROSPERO) under registration number CRD420261412813.

### 2.2. Eligibility Criteria and PICO Framework

The research focused on evaluating the efficacy and safety of TV-based therapies. The PICO model was used to structure and focus the literature review.

- Population: The analysis included adult female patients who had been diagnosed with advanced, recurrent, or metastatic cervical cancer.
- Intervention: TV administered either as a monotherapy or in combination with other therapeutic agents (e.g., immunotherapy or chemotherapy).
- Comparison: Standard chemotherapy (investigator’s choice monotherapy) or other standard-of-care regimens, where applicable. For single-arm trial cohorts, no direct comparator was required.
- Outcome: Focused on two key aspects: clinical efficacy and safety profile. Antitumor efficacy was assessed based on the objective response rate (ORR) according to RECIST 1.1 criteria (including the rates of complete response [CR] and partial [PR]), disease control rate (DCR), progression-free survival (PFS), overall survival (OS), and duration of response (DOR). The safety profile was evaluated by analysing the incidence and severity of treatment-emergent adverse events (TEAEs) and serious adverse events (SAEs), with particular emphasis on events of special clinical significance (AESIs), which included ophthalmic complications, peripheral neuropathy, and bleeding events, classified according to the CTCAE scale. Health-related quality of life (QoL) and patient-reported outcomes were assessed, where available, using validated instruments, including the EORTC QLQ-C30, the cervical cancer-specific EORTC QLQ-CX24 module, and the EQ-5D-5L questionnaire. Extracted PRO measures included baseline and follow-up questionnaire completion rates, mean changes from baseline, and the proportion of patients achieving a clinically meaningful improvement or deterioration.

Studies published in English between 2019 and 2026 were eligible for inclusion. The review considered prospective clinical trials (phase I–III) evaluating TV as monotherapy or in combination with immunotherapy in adult patients with CC.

In addition to primary trial reports, secondary publications derived from the same parent clinical trial programmemes—including predefined subgroup analyses, PROs, and extended follow-up reports—were also included when they provided additional clinically relevant efficacy or safety data. Multiple publications originating from the same trial were treated as linked records and synthesised under a single trial program.

Exclusion criteria comprised review articles, preclinical studies (animal or cellular models), conference abstracts or posters without complete data, and publications lacking relevant efficacy or safety outcomes related to TV therapy. Conference abstracts and posters were excluded only where they did not contain sufficiently comprehensive data on efficacy or safety. The risk of publication bias was minimised by searching ClinicalTrials.gov and reviewing the reference lists of eligible studies.

### 2.3. Search and Selection Process

**Figure 1:**
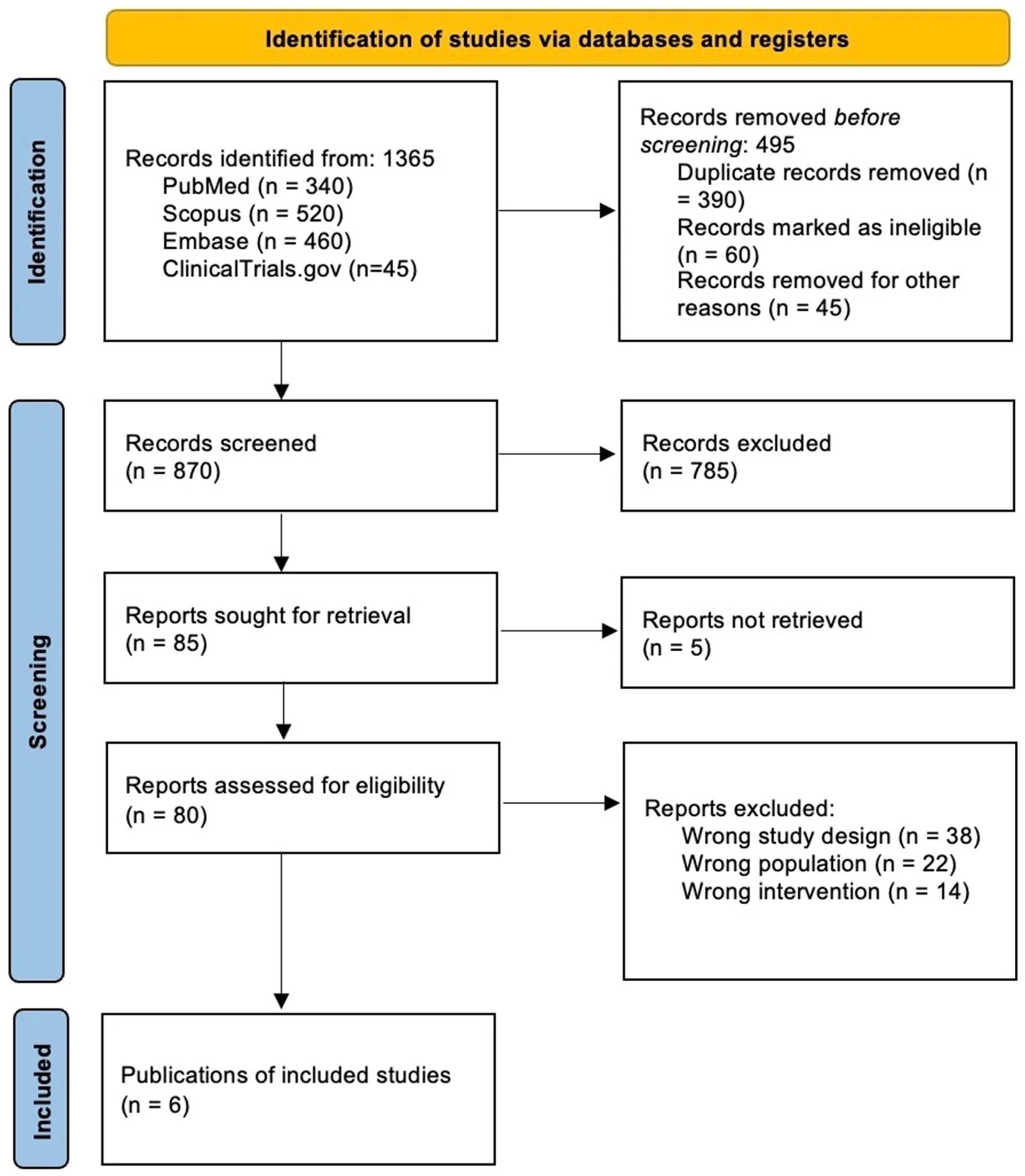
PRISMA 2020 flow diagram of the identification, screening, eligibility assessment, and inclusion of studies. n = number of records/reports at each stage. “Records removed before screening” (n = 495) comprised duplicate records identified through deduplication (n = 390), records flagged as ineligible on the basis of publication type or language (n = 60), and records removed for other reasons (n = 45)-specifically 25 non-English-language publications, 12 editorials, letters, or commentaries, and 8 records lacking an available abstract, all identified during reference import prior to formal screening. “Records excluded” (n = 785) refers to records excluded at the title/abstract screening stage against the eligibility criteria in Section 2.2; “Reports excluded” (n = 74) reflects full-text exclusions for wrong study design (n = 38), wrong population (n = 22), or wrong intervention (n = 14).

A systematic search of PubMed, Scopus, Embase, and ClinicalTrials.gov was performed to identify relevant studies published between 1 January 2019, and 1 April 2026. The final search was conducted on 25 April 2026. The following keywords were used with database-specific adaptations: (“Tisotumab Vedotin” OR “Tivdak” OR “HuMax-TF-ADC”) AND (“Cervical Cancer” OR “Uterine Cervical Neoplasms”) AND (“Antibody–drug conjugate” OR “innovaTV”). The complete, database-specific search strings were as follows: PubMed-(“Tisotumab Vedotin”[tiab] OR “Tivdak”[tiab] OR “HuMax-TFADC”[tiab]) AND (“Uterine Cervical Neoplasms”[MeSH Terms] OR “Cervical Cancer”[tiab]) AND (“Immunoconjugates”[MeSH Terms] OR “Antibody-drug conjugate”[tiab] OR “innovaTV”[tiab]); Scopus-((“Tisotumab Vedotin” OR “Tivdak” OR “HuMax-TF-ADC”) AND (“Cervical Cancer” OR “Uterine Cervical Neoplasms”) AND (“Antibody–drug conjugate” OR “innovaTV”)); Embase-(‘tisotumab vedotin’/exp OR ‘tisotumab vedotin’ OR ‘tivdak’ OR ‘humax-tf-adc’) AND (‘uterine cervix carcinoma’/exp OR ‘cervical cancer’) AND (‘antibody drug conjugate’/exp OR ‘innovatv’); ClinicalTrials.gov -“Cervical Cancer”, “Tisotumab Vedotin” with status filters set to include completed, active, and recruiting studies. To ensure completeness, a manual search of the reference lists of all eligible studies was also performed. Three researchers (N.G., N.J. and Z.D.) independently screened titles, abstracts, and full-text articles using predefined eligibility criteria. Screening decisions and the reason for exclusion at each stage were logged for every record using a standardised eligibility checklist rather than dedicated screening software. Any discrepancies were resolved through discussion with two additional reviewers (J.P. and N.P.). Furthermore, the unanimous agreement of all four reviewers was required before a final decision was made on whether to include or exclude a study. ClinicalTrials.gov records were cross-checked against peer-reviewed publications to avoid duplicate inclusion of registry entries and published reports. When multiple publications originated from the same parent clinical trial, these were treated as linked records and synthesised under a single trial programme. The database search identified 1365 records. Of these, 495 records were removed before screening (including duplicates and records marked as ineligible by automation tools). Duplicates were removed manually. The retrieved records were compared against each other in terms of author surname, article title, journal and year of publication. Duplicate entries were removed following a joint decision by the reviewers conducting the selection. No software was used for the automatic removal of duplicates. Following the screening of 870 records, 785 were excluded. After accounting for 5 reports not retrieved, 80 reports were assessed for eligibility, of which 74 were excluded due to wrong study design, population, or intervention. Ultimately, six publications representing four unique clinical trial programmes were included in the final qualitative synthesis. The study selection process, including identification, screening, eligibility, and inclusion, is presented in the PRISMA flow diagram in Figure 1.

### 2.4. Data Extraction

The review included six publications derived from four major clinical trial programmes (innovaTV 201, innovaTV 204, innovaTV 205, and innovaTV 301). These publications collectively reported data from 548 unique patients, with linked secondary reports providing additional subgroup, long-term follow-up, and PRO data.

Data extraction was performed independently by two reviewers using a standardised form, with any discrepancies resolved through discussion or consultation with a third reviewer. Extracted variables included parent trial programme, publication type (primary efficacy, subgroup, follow-up, or PRO report), study population, treatment regimen, and efficacy and safety outcomes. Specifically, extracted data encompassed study characteristics (author, year, study design), patient demographics (total number of patients, age, gender), intervention details (TV dosage, regimen, monotherapy vs. combination therapy), and outcome measures related to efficacy (ORR, PFS, OS, DoR, CR, PR, SD, PD, Clinical Benefit [CB]) and safety (incidence and severity of TRAEs).

Patient-reported outcome data, including the PRO instrument used, assessment time points, questionnaire completion rates, changes from baseline, and thresholds for clinically meaningful change, were also extracted.

### 2.5. Assessment of Risk of Bias in the Included Studies

**Figure 2:**
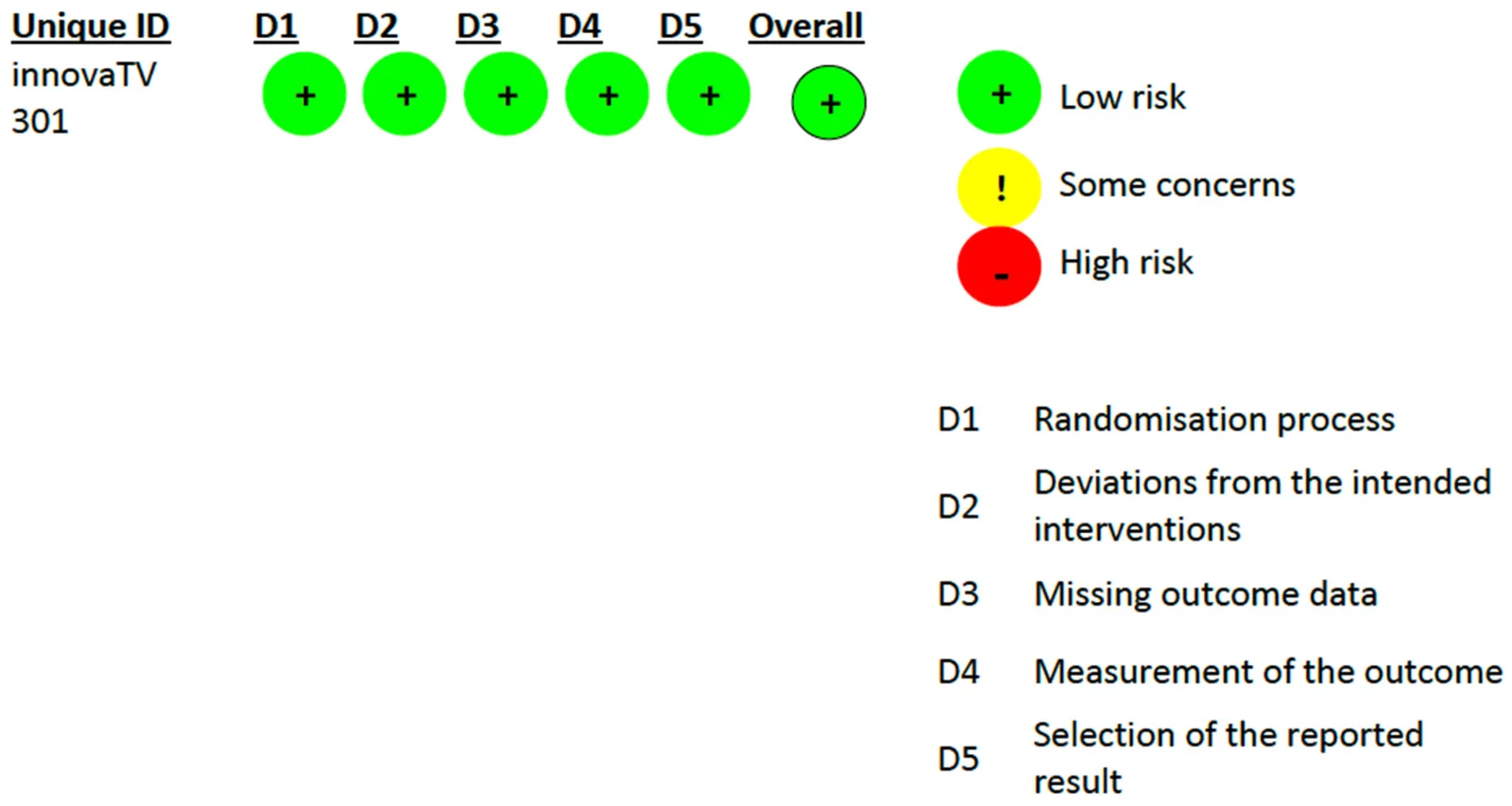
Risk of bias assessment for the innovaTV 301 trial (Cochrane RoB 2 tool).

Risk of bias was assessed at the level of the parent randomised trials using the updated Cochrane Risk of Bias tool for randomised trials (RoB 2.0) [14]. This tool evaluates five domains: (1) bias arising from the randomization process, (2) bias due to deviations from intended interventions, (3) bias related to missing outcome data, (4) bias in outcome measurement, and (5) bias in the selection of reported results. Two independent reviewers applied RoB 2.0 to all parent trials, recording supporting evidence and providing judgments of low risk, high risk, or some concerns for each domain. Discrepancies were resolved through discussion or adjudication by a third reviewer. For linked secondary publications (such as subgroup, PRO, and long-term follow-up reports), risk-of-bias judgments were anchored to the corresponding parent trial, with additional consideration of reporting completeness and outcome-specific limitations. The domain-level RoB 2.0 summary for all parent randomised studies included in the review is shown in Figure 2. Based on this assessment, the innovaTV 301 trial was judged to have an overall low risk of bias, as it demonstrated a low risk of bias across all five evaluated domains.

For the single-arm, non-randomised studies (innovaTV 201, 204, and 205), the methodological quality was evaluated using the NIH Quality Assessment Tool for Before-After (Pre-Post) Studies with No Control Group. This tool was selected to address the specific design characteristics of early-phase oncology trials. Key criteria, such as population definition, clarity of intervention, and objectivity of outcome measurement, were assessed. While these trials were inherently “open- label”—a standard design in early-phase oncology that precludes blinding—the risk of detection bias was mitigated by the use of strictly defined, radiologically based RECIST 1.1 criteria for tumour response assessment. This ensures that efficacy outcomes remain objective and reproducible. These studies were consistently rated as having an overall quality of “Good,” indicating high reliability of the reported clinical outcomes within their respective trial design contexts.

This tailored approach to risk of bias assessment, specific to each study design, ensured a comprehensive evaluation of study quality and enhanced the overall reliability of the review findings.

## 3. Results

### 3.1. Mechanism of Action of the Drug

TV is the first ADC to be used, with a drug that targets TF on the surface of cancer cells. It consists entirely of a human monoclonal antibody specific for TF, conjugated with the cytotoxic agent MMAE. MMAE disrupts microtubules and kills actively dividing cancer cells in the G2/M phase and induces apoptosis in those that have internalised TV. As MMAE has the ability to penetrate cell membranes, it can diffuse out of the cell into the tumour microenvironment, where it can induce the death of neighbouring cancer cells in the division phase. This anti-tumour activity is further enhanced by the ability of TV to bind to FcγRIIIa on neighbouring NK cells. This leads to antibody-dependent cellular cytotoxicity in TF(+) tumour cells. The antibody and MMAE are linked via a valine-citrulline linker, which is proteolytically cleaved and released following internalisation of TV into cancer cells [15,16].

The primary role of TF as a transmembrane glycoprotein is to initiate the coagulation cascade and it exhibits signalling properties. However, during the process of oncogenesis, TF plays an important role in tumour angiogenesis, progression and the formation of tumour metastases. This is possible through the utilisation of an intracellular signalling cascade activated by the protease-activating receptor 2 via the early binding of FVIIa to TF. TF is abnormally expressed in many types of cancer, including non-small cell lung cancer (33–88% TF(+) detection rate), endometrial cancer (14–100% TF(+) detection rate), prostate cancer (47–75% TF(+) detection rate), ovarian cancer (75–100% TF(+) detection rate) and cervical cancer (100%), which is often associated with poor clinical outcomes. TF expression is upregulated in tumours through oncogenic events such as constitutive activation of the MAPK and PI3K signalling pathways, hypoxia-induced signalling and loss of tumour suppressor genes [15,16,17].

**Figure 3:**
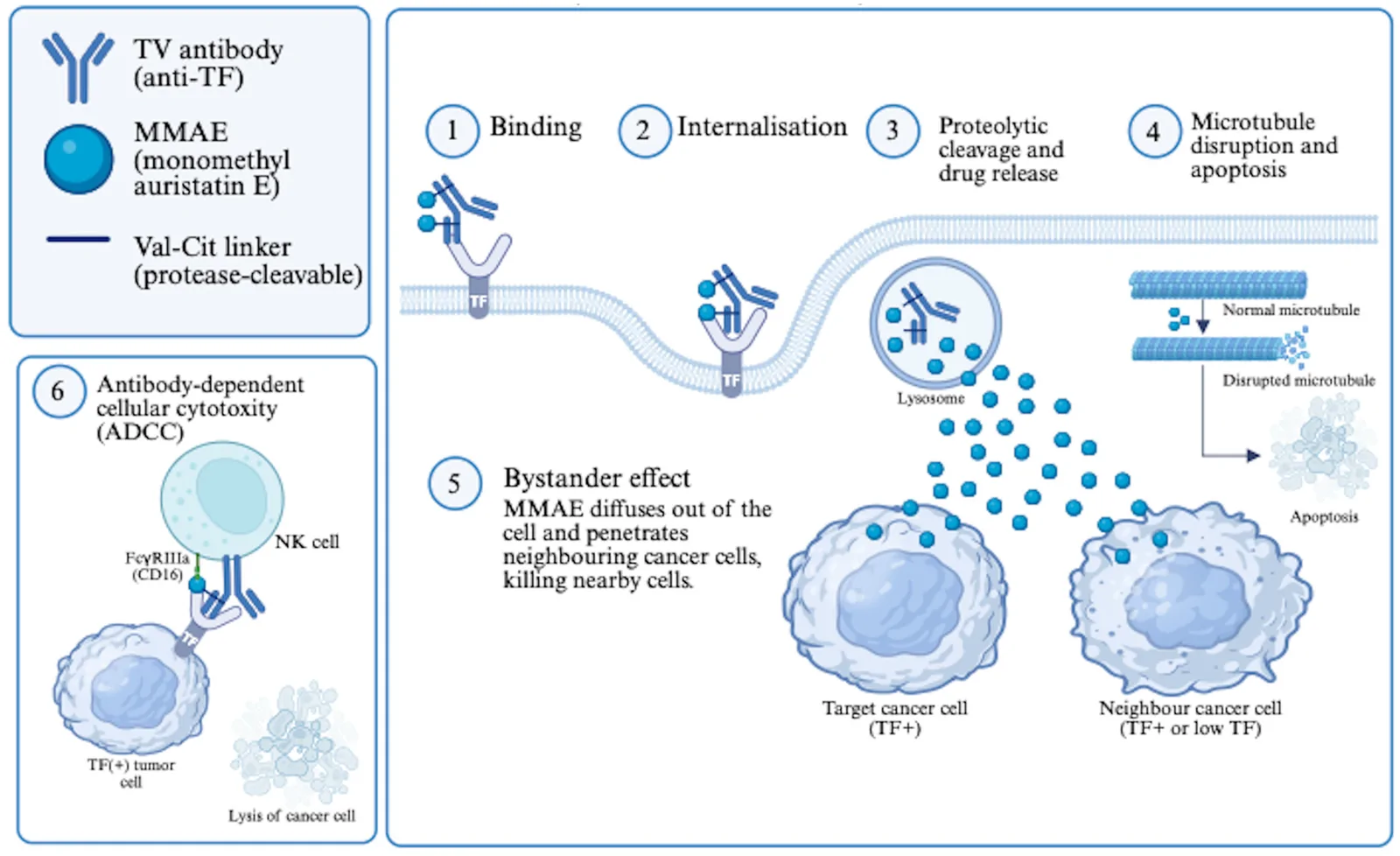
Mechanism of action of TV (Tisotumab Vetodin).

The TV antibody was selected from a panel of human monoclonal antibodies specific for TF due to its high affinity and ability to disrupt intracellular signalling of the protease-activated receptor 2 [16]. Furthermore, it was found that TV inhibits TF- activated factor VII-dependent intracellular signalling, with minimal effect on coagulation activity and interleukin-8 production [17]. The mechanism of the drug is presented in Figure 3.

### 3.2. Clinical Efficacy Outcomes

The analysis of the clinical efficacy of TV in the treatment of cervical cancer encompasses four key clinical trial programmes: innovaTV 201, innovaTV 204, innovaTV 205 and innovaTV 301 [15,16,17,18,19,20]. The primary endpoints assessed in these trials, which determined the therapeutic benefits of the conjugate, were the objective response rate (ORR) assessed according to RECIST 1.1 criteria, the disease control rate (DCR, Disease Control Rate), progression-free survival (PFS, Progression-Free Survival), overall survival (OS, Overall Survival) and duration of response (DOR, Duration of Response).

A detailed summary of the key clinical parameters being compared is provided in Table 1.

The efficacy-evaluable population comprised 32 patients in arm E and 34 patients in arm F. The corresponding treated/safety populations comprised 33 and 35 patients, respectively. One patient in arm E received brentuximab and was excluded from the efficacy analysis, while one patient in arm F had no measurable disease at baseline and was therefore excluded from the efficacy analysis.

#### 3.2.1. Antitumour Activity in Monotherapy

Preliminary evidence of the biological efficacy of TV therapy in a population of patients with disease recurrence following first-line chemotherapy was provided by the innovaTV 201 trial (N = 55). The overall response rate (ORR) and duration of response (DOR) were assessed separately by an Independent Review Committee (95% CI: 12–35%) and by the investigators (95% CI: 13–37%) (Table 1) [17].

A subsequent, single-arm Phase II trial, innovaTV 204 (N = 101), showed a similar overall response rate (ORR) (95% CI: 16–33%). Meanwhile, the complete response (CR) rate was 7%, with the remainder being partial responses (PR). The disease control rate (DCR), duration of response (DOR), progression-free survival (PFS) and overall survival (OS) for this study are presented in Table 1, together with 95% confidence intervals of 62–81% (DCR), 4.2–NR months (DOR), 3.0–5.4 months (PFS) and 9.6–13.9 months (OS) [18].

The efficacy of TV monotherapy compared to standard second- or third-line single-agent chemotherapy was directly confirmed in the international, randomised Phase III innovaTV 301 trial. A total of 502 patients were enrolled in the trial (TV group: N = 253, control group: 249). The overall response rate (ORR) was significantly higher in the TV arm (95% CI: 13.3–23.1%; n = 45/253) than in the control arm, which achieved an ORR of 5.2% (95% CI: 2.8–8.8%; n = 13/249) (odds ratio: 3.96; *p* < 0.0001) (Table 1). In the treatment group, 2.4% of patients achieved complete remission (CR) (n = 6), compared to 0% (n = 0) in the control group. The proportion of patients who achieved complete or partial remission (DCR) was 58.2% in the control group [19].

With regard to the primary endpoints of the innovaTV 301 trial, overall survival (OS) in the TV group was 9.8–14.9 months (95% CI) and was significantly longer than in the group receiving chemotherapy (95% CI: 7.9–10.7 months). This corresponds to a hazard ratio (HR) for death of 0.70 (95% CI: 0.54–0.89; *p* = 0.0038) (Table 1). Progression-free survival (PFS) in the TV group was 4.0–4.4 months (95% CI), compared to 2.6–3.2 months in the control group (HR = 0.67; 95% CI: 0.54–0.82; *p* < 0.0001). The median duration of response (DOR) was 4.8 months in the control group, compared to the value for the TV group given in Table 1 [19].

#### 3.2.2. New Therapeutic Perspectives in Combination Regimens

The open-label, multicentre Phase Ib/II innovaTV 205 trial evaluated the efficacy of TV in combination with immune checkpoint inhibitors (pembrolizumab) and platinum-based chemotherapy (carboplatin). The results were analysed by cohort, taking into account the treatment line and the type of regimen used [20].

First-line treatment (1L)

In the population of patients previously untreated for cervical cancer, therapeutic efficacy was assessed in three separate study arms:

Arm E (efficacy population, n = 32): TV plus pembrolizumab resulted in a confirmed ORR of 40.6% (13/32; 95% CI: 23.7–59.4%). The median PFS was 5.3 months (95% CI: 4.0–12.2), the median OS was 30.7 months (95% CI: 13.0–NR), and the median DOR had not been reached (95% CI: 3.0–NR) at the time of the updated analysis [20]. The median progression-free survival (PFS) was 5.3 months (95% CI: 4.0–11.0). The median overall survival (OS) was 30.7 months (95% CI: 16.9–NR). The median duration of response (DOR) had not been reached (NR; 95% CI: 4.2–NR) during the reported follow-up [20].

Arm D (N = 33) TV+ carboplatin: The ORR was 54.4% (N = 18/33; 95% CI: 36.4–71.9%). The median progression-free survival (PFS) in this cohort was 6.9 months (95% CI: 5.1–11.3). The median overall survival (OS) reached 25.5 months (95% CI: 17.2–NR). The median duration of response (DOR) was 8.6 months (95% CI: 4.2–NR).

Arm H (N = 38) TV+ carboplatin+pembrolizumab: In this arm, an ORR of 65.8% was observed (N= 25/38; 95% CI: 48.6–80.4%). The median overall survival (OS) was 28 months (95% CI: 21.3–NR), with a median DOR of 13.3 months (95% CI: 5.9–NR) [20].

Second- and third-line treatment (2L/3L)

The efficacy of combining TV with immunotherapy in patients who had previously received systemic treatment was assessed in arm F of the study. Arm F (efficacy population, n = 34): TV plus pembrolizumab resulted in a confirmed ORR of 35.3% (12/34; 95% CI: 19.7–53.5%). The median PFS was 5.6 months, the median OS was 15.3 months, and the median DOR was 18.2 months in the updated analysis [20].

### 3.3. Toxicity and Safety Profile

Data on the toxicity and safety profile of TV were evaluated on the basis of four main clinical trials: innovaTV 301, innovaTV 204, innovaTV 201 and innovaTV 205 [15,16,17,18,19,20].

The study by Yonemori et al. as part of the multicentre innovaTV 301/ENGOT-cx12/GOG-3057 assessed the safety and adverse effects of TV [19]. The median duration of treatment exposure in the TV group was 3.5 months (range 0.7–11.0) compared with 1.9 months (range 0.3–13.6) for chemotherapy exposure. Grade ≥ 3 adverse events occurred in 66.0% of the chemotherapy group, compared with the TV group rate reported in Table 2. The most commonly reported adverse events of any grade in the TV group in this study were conjunctivitis (46.9%), nausea (38.8%) and peripheral sensory neuropathy (36.7%). Other reported TEAEs included: alopecia (30.6%), epistaxis (24.5%), decreased appetite (24.5%), fever (24.5%), anaemia (16.3%), neutropenia (8.2%), decreased neutrophil count (2.0%) and diarrhoea (6.1%). Serious adverse events (SAEs) occurred in 14.3% of patients receiving TV (compared with 34% of patients in the chemotherapy group), although no fatal adverse events (Grade 5) were reported in either group [20].

Particular attention was paid to specific toxicity groups, which included ophthalmic complications (53.1%), peripheral neuropathy (38.8%) and bleeding events (42.9%), which were managed in 92.3%, 47.4% and 61.9% of patients, respectively [20].

The adverse events (TEAEs) observed in this study necessitated a dosage adjustment in 44.9% of patients treated with TV. In contrast, complete and premature discontinuation of therapy in the TV-treated group was recorded in 6 patients (12.2%) due to peripheral sensory neuropathy (n = 2; grade 1 and 2), conjunctivitis (n = 1; grade 2), corneal degeneration (n = 1; grade 3), gait disturbances (n = 1; grade 2) and Stevens–Johnson syndrome (SJS) (n = 1), which was a disadvantage of this therapy compared with chemotherapy, where no discontinuation of therapy due to adverse effects was reported [20].

It is worth noting that throughout the study, one case of grade 3 SJS was reported in each of the treatment arms. In the case of TV treatment, the SJS case occurred on day 24 of treatment (during the second cycle) and led to the patient’s withdrawal from the study, and the symptoms of toxicity had not been controlled by the time of withdrawal [20].

Another key study included in this review is the international, multicentre phase II clinical trial innovaTV 204/GOG-3023/ENGOT-cx6 published by Coleman et al. [18]. The median duration of TV treatment in this trial was 4.2 months (IQR 2.5–5.5). In the analysed study group, TEAEs of any grade occurred in 92% of patients. In turn, grade ≥ 3 TEAEs accounted for 28% of all adverse events. The most commonly reported were: alopecia (38%), epistaxis (30%), nausea (27%), conjunctivitis (26%), fatigue (24%) and dry eye syndrome (23%). Serious adverse events (SAEs) occurred in 13% of participants. During safety monitoring, one treatment-related death (septic shock on day 5 after the start of therapy) and three deaths unrelated to drug administration were also recorded [18].

The TV safety analysis also assessed in detail toxicity associated with ophthalmic complications (Table 3), the most common of which were conjunctivitis (26%), dry eye syndrome (23%) and keratitis (11%). Peripheral neuropathy was observed in a total of 33% of patients, whilst bleeding events occurred in 39%. The most common types of bleeding were epistaxis, genital haemorrhage and haematuria, with the vast majority (90%) of these adverse events being mild in nature and successfully managed [18].

The occurrence of adverse events in this study also led to changes in the treatment regimen, with dose reduction, dose suspension/omission, and complete discontinuation rates detailed in Table 2 [18].

The results of the first phase I-II clinical trial in humans, InnovaTV 201, conducted by Bono et al. [16], formed the basis for subsequent analyses. The toxicity and safety profile in the solid tumour patient group was predictable, and although TEAEs occurred in the majority of study participants, they were manageable. The most common adverse events of any grade included: nose bleeds (69%), fatigue (56%), nausea (52%), alopecia (44%), conjunctivitis (43%), decreased appetite (36%), constipation (35%), diarrhoea (30%), vomiting (29%), peripheral neuropathy (22%), dry eyes (22%) and abdominal pain (20%). Grade ≥ 3 adverse events occurred in 56% of patients and were most commonly fatigue, anaemia, abdominal pain, hypokalaemia, conjunctivitis, hyponatraemia and vomiting. Treatment-related SAEs occurred in 46% of patients, the most common of which were vomiting (4%), abdominal pain (3%) and anaemia (3%) [16].

Crucial to the analysis of the results is the updating and refinement of data based on the study by Hong et al. presenting results for a population with recurrent or metastatic cervical cancer [17]. In this group, the safety profile was similar to that of the overall study population from the previous study. The most common adverse events of any TV grade were epistaxis (51%), fatigue (51%), nausea (49%), conjunctivitis (42%) and alopecia (40%). Grade ≥ 3 adverse events occurred in 56% of patients with cervical cancer, the most common of which was anaemia (11%) [17].

Treatment discontinuation due to adverse events occurred in 22% of patients overall, whereas in the cervical cancer group this proportion was lower at 18%, with the main cause of discontinuation being peripheral neuropathy (9%) [16,17]. Additionally, 12% of patients overall, and 9% in the cervical cancer group, experienced an infusion-related event, namely abdominal pain and vomiting, both occurring in 2% of patients [16,17]. During the dose-escalation phase of the innovaTV201 trial, three of 27 patients (11%) experienced toxicity resulting in dose reduction (type 2 diabetes, mucositis and neutropenic fever; grade 3). Overall and cervical-cancer-specific dose reduction rates are reported in Table 2 [17].

In the study by Bono et al., adverse events of special clinical significance (AESIs) were also subjected to detailed analysis; these included haemorrhagic events, neuropathy and ophthalmic complications (including conjunctivitis, ulcers, keratitis and conjunctival adhesions) with overall incidence reported in Table 3. The most commonly reported haemorrhagic complication was epistaxis, with as many as 98% (100 out of 102) of these cases being mild (Grade 1). Neuropathy of any severity occurred in 43% of patients (63 out of 147), of whom 7% (10 patients) had grade ≥ 3 neuropathy. The median time to onset of neuropathic symptoms was 8.7 weeks (range: 3.0–14.1). In the group of patients with neuropathy symptoms, 81% (51 out of 63 patients) had previously undergone taxane-based chemotherapy (most commonly paclitaxel). By the time of the analysis, complete resolution of neuropathy symptoms had been observed in 16% of patients (10 out of 63). Ophthalmic events of any severity occurred in 60% of patients (88 out of 147), with conjunctivitis observed in 43% of all patients. Serious ocular adverse events were reported in 7% of patients (11 patients). Importantly, the implementation of appropriate preventive and palliative strategies led to a significant reduction in the incidence of conjunctivitis—this proportion fell from 56% (43 out of 77 patients) to 29% (20 out of 70 patients) [17].

By comparison, in patients with cervical cancer, an analysis of the same adverse events of particular clinical significance (Table 3) revealed no haemorrhagic events of grade ≥3, and peripheral neuropathy was predominantly mild (any grade 36%; grade 3 4%). Ophthalmic complications occurred in as many as 65% of patients. Crucially, the study by Hong et al. confirmed the efficacy of the evolving eye-protection protocol: structured adherence reduced this proportion from 80% to 60%, with the incidence of conjunctivitis reduced from 80% to 28% [17].

Regarding mortality, three deaths (11%) were recorded during the dose-escalation phase; however, none of these were related to the toxicity of the investigational drug (two patients died due to cancer progression, and one patient with head and neck squamous cell carcinoma died due to haemorrhage from a throat tumour). During the dose-expansion phase, there were 6 deaths (4%), of which 5 were directly due to tumour progression, and one death from pneumonia was considered by the investigators to be potentially related to TV administration [16]. In a subsequent update of the study, which was expanded to include a larger group of patients with cervical cancer, 2 deaths were reported, mainly related to disease progression [17].

The evolution of treatment regimens using TV from monotherapy to advanced multi-drug combinations is reflected in the long-term, 5-year results of the multicentre phase Ib/II clinical trial innovaTV 205/ENGOT-cx8/GOG-3024, published by Van Nieuwenhuysen et al. [20]. The aim of this analysis was to evaluate the efficacy and safety profile of TV in combination with other standard therapeutic agents in patients with recurrent or metastatic cervical cancer [20].

Long-term assessment of the safety profile in this case showed that toxicity in combination with other drugs was consistent with the safety profiles of the individual drugs, and no new concerning side effects were reported. The most common general symptoms of any grade across the entire study population were nausea, diarrhoea, anaemia, alopecia and fatigue. Grade ≥ 3 treatmentrelated AE rates by arm are reported in Table 2. In study group H, characterised by the most intensive treatment regimen, the most common grade ≥ 3 AEs were: anaemia (39.5%), thrombocytopenia (15.8%), fatigue (13.2%) and neutropenia (13.2%). Complete discontinuation rates by arm are also reported in Table 2. The highest rate, in arm H, was primarily driven by cumulative peripheral neuropathy [20].

In the context of TV-specific AESIs (ophthalmic complications, peripheral neuropathy, and bleeding), the addition of further drugs did not alter their overall nature, although it did affect their frequency by arm, as shown in Table 3. Grade 4 treatment-related bleeding events occurred in one patient (2.9%) in arm F (haematuria and haemorrhagic cystitis—related to TV) and two patients (5.3%) in arm H (arterial haemorrhage, related to TV; gastric haemorrhage, related to TV and bevacizumab). One patient (3.0%) in arm E experienced a Grade 5 AE bleeding event [20].

Table 2 and Table 3 below summarises the toxicity and safety profile of TV based on data analysed from four leading clinical trials

## 4. Discussion

TV is an innovative drug offering hope to patients with recurrent or metastatic CC. It is the first ADC to be approved by the FDA for use in treating these patients. In clinical trials, it demonstrated very good efficacy in patients who had previously undergone treatment, achieving an ORR of 17.8–54.5% depending on the trial conducted.

The initial innovaTV 201 trial demonstrated optimal dosing, safety and activity of the drug. In turn, the innovaTV 204 trial showed an ORR of 24%, which enabled the FDA to begin the registration process for TV. The innovaTV 301 trial provided definitive confirmation of better outcomes in patients receiving TV, demonstrating an extension of overall survival (OS). In turn, the innovaTV 205 trial demonstrated the potential for using TV in earlier stages of the disease. These studies have shown that the drug works extremely well in combination with carboplatin, immunotherapy or bevacizumab, which means it has the potential to transform the entire course of treatment.

### 4.1. Interpretation of the Efficacy of TV

Analysis of the results included in this systematic review clearly indicates significant clinical activity of TV in patients with cervical cancer. To fully understand its therapeutic role, the efficacy rates obtained should be compared with historical registry data for standard chemotherapy and immune checkpoint inhibitors (ICI) [4,21].

#### 4.1.1. Comparison with Second-Line Monochemotherapy

For decades, the prognosis for patients showing progression after first-line chemotherapy, based on platinum derivatives and paclitaxel with the possible addition of bevacizumab, remained extremely poor. Second-line chemotherapy, i.e., tamoxifen, vinorelbine, gemcitabine and irinotecan, therefore remained the standard of care [4,21].

Historical data for these cytostatic agents reported an ORR of merely 5–12%, with median overall survival (OS) rarely exceeding 6 to 9 months. The most direct validation in this regard was provided by the randomised phase III innovaTV 301 trial included in the review [19].

Against this background, TV monotherapy demonstrates a clinical advantage. ORR rates of 17.8% in the innovaTV 301 trial and as high as 24% in the innovaTV 204 trial represent at least a twofold increase in the chances of achieving an objective anti-tumour response. Crucially, from the perspective of patient benefit, this translated into statistically and clinically significant prolongation of median OS to 11.5–12.1 months, which in this patient population had previously been a difficult barrier to overcome with monotherapy [18,19].

#### 4.1.2. Comparison of TV with Immunotherapy (Pembrolizumab) as Monotherapy

The introduction of PD-1 checkpoint inhibitors has revolutionised gynaecological oncology. In the context of second-line treatment, the KEYNOTE-15 trial has become the benchmark, leading to the registration of pembrolizumab for PD-1-expressing cervical cancer (CPS ≥ 1).

In the KEYNOTE-158 trial, pembrolizumab achieved an ORR of 14.6% (95% CI: 7.8–24.2%) in the overall population and 17.1% in PD-1-positive patients. The median OS was 11 months [22].

When comparing these values to the results of TV in the innovaTV 204 and innovaTV 301 trials, the conjugate demonstrates similar, and in some cohorts numerically higher and faster, response dynamics. The main advantage of TV over pembrolizumab as monotherapy, however, lies in its independence from the status of immune biomarkers. Pembrolizumab shows minimal efficacy in PD-1-negative patients (CPS ≤ 1) (ORR close to 0%). In contrast, TV, as a molecularly targeted drug against the tumour factor (TF), exhibits high expression stability in cervical cancer, thereby effectively inducing cell death even in patients who did not benefit from pembrolizumab [23].

#### 4.1.3. Synergy in Combination Regimens

The most promising direction for interpreting the results is indicated by data from the innovaTV 205 trial, which evaluated TV in first-line (1L) treatment in combination with chemotherapy or pembrolizumab [20]. The current standard of first-line treatment (platinum + paclitaxel + pembrolizumab ± bevacizumab) has established a median PFS of approximately 10.4 months [24].

The results from arm H in the innovaTV 205 trial (TV + carboplatin + pembrolizumab), which achieved an ORR of 65.8% and a median OS of 28 months, suggest that the inclusion of TV in first-line treatment will enable deeper therapeutic responses to be achieved [20].

In summary, TV significantly redefines the treatment algorithm for cervical cancer. As a monotherapy, it represents an effective alternative to the less effective conventional chemotherapy in patients after disease progression, whilst its unique mechanism of action provides a biological basis for the use of this drug in multi-agent regimens in first-line treatment.

#### 4.1.4. Factors Contributing to Variability in Efficacy Across the innovaTV Trials

Although all four innovaTV programmes demonstrated clinically relevant antitumour activity of TV, numerical differences in ORR, PFS, OS, and DOR should not be interpreted as direct evidence of the superiority of one regimen over another. The studies differed substantially in treatment setting, previous treatment exposure, trial design, sample size, regimen composition, response assessment, and duration of follow-up. Consequently, cross-trial comparisons remain descriptive and hypothesis-generating rather than confirmatory [15,16,17,18,19,20].

First, the line of treatment and the extent of previous systemic therapy are likely to have influenced the observed outcomes. The cervical cancer expansion cohort of the phase I/II innovaTV 201 programme and the phase II innovaTV 204 study evaluated TV monotherapy in patients with previously treated recurrent or metastatic cervical cancer [16,17,18]. Despite differences in study phase and sample size, both studies reported consistent ORRs of approximately 22–24%, supporting reproducible single-agent activity in previously treated disease [17,18]. In innovaTV 204, the median PFS was 4.2 months, the median OS was 12.1 months, and the median DOR was 8.3 months [18]. In the cervical cancer cohort of innovaTV 201, the ORR was 22% according to independent review and 24% according to investigator assessment, while the median DOR was 6.0 and 4.2 months, respectively [17].

The numerically lower ORR of 17.8% observed with TV in innovaTV 301 should not be interpreted as evidence of reduced biological activity. Unlike the earlier single-arm studies, innovaTV 301 was a large, randomised phase III trial in which 502 patients receiving second- or third-line treatment were assigned to TV or investigator’s-choice chemotherapy. TV significantly improved OS and PFS and produced a higher ORR than chemotherapy [19]. The hazard ratios for death and disease progression provide stronger comparative evidence of treatment benefit than numerical comparisons of ORR between unrelated single-arm cohorts. Moreover, the innovaTV 301 population reflected a contemporary and heterogeneous treatment history: 63.9% of patients had previously received bevacizumab and 27.5% had received an anti–PD-1 or anti–PD-L1 agent [19]. Differences between innovaTV 204 and innovaTV 301 may therefore reflect not only study design and sample size but also differences in previous treatment exposure and baseline disease characteristics.

Second, the higher response rates reported in innovaTV 205 were observed predominantly with combination regimens and, in several cohorts, in the first-line setting. Patients in arms D, E, and H had not received prior systemic therapy for recurrent or metastatic disease, whereas patients in arm F had experienced disease progression during or after one or two previous lines of systemic treatment. In addition, patients enrolled in the pembrolizumab-containing arms E, F, and H had not previously received anti–PD-1 or anti–PD-L1 therapy [20]. These eligibility criteria may have contributed to differences in treatment responsiveness between the innovaTV 205 cohorts and the later-line monotherapy studies.

The confirmed ORR was 54.5% with first-line TV plus carboplatin in arm D, 40.6% with first-line TV plus pembrolizumab in arm E, and 35.3% with second- or third-line TV plus pembrolizumab in arm F. The highest ORR, 65.8%, was observed in arm H with first-line TV plus carboplatin and pembrolizumab, with or without bevacizumab [20]. However, bevacizumab was administered to 19 of the 38 patients in arm H, creating additional within-cohort treatment heterogeneity. The observed response rate therefore cannot be attributed exclusively to the triplet regimen or to any individual treatment component [20].

The within-programme findings suggest that both treatment line and regimen composition may contribute to differences in efficacy. Patients receiving first-line treatment are less heavily pretreated than patients with disease progressing after several systemic regimens, while chemotherapy and immune-checkpoint inhibition may provide complementary antitumour activity when combined with TF-directed delivery of MMAE. Nevertheless, these explanations remain hypotheses. The innovaTV 205 cohorts were small, non-randomised, and not designed to provide direct comparisons between treatment arms; therefore, the independent contribution of TV, carboplatin, pembrolizumab, and bevacizumab cannot be determined conclusively [20].

Third, differences in trial design and sample size affect the precision and interpretation of the efficacy estimates. InnovaTV 201 and innovaTV 204 were open-label, single-arm studies, while innovaTV 205 included several small, non-randomised dose-expansion cohorts [16,17,18,20]. Such designs are appropriate for identifying preliminary antitumour activity, but are susceptible to patient-selection effects and cannot reliably separate treatment effects from differences in baseline prognosis. The innovaTV 205 cohorts included only 33–38 enrolled patients per arm, and several efficacy estimates were accompanied by wide confidence intervals [20]. By comparison, innovaTV 301 included a concurrent chemotherapy control group and was powered to evaluate survival outcomes, providing the most robust comparative evidence for TV in previously treated recurrent or metastatic cervical cancer [19].

Finally, differences in response assessment and follow-up maturity also contributed to heterogeneity. In innovaTV 201, ORR and DOR differed depending on whether tumour response was assessed by an independent review committee or by investigators [17]. In innovaTV 205, the median DOR remained not reached in arm E, while several survival estimates had wide confidence intervals. Follow-up also differed across the innovaTV 205 cohorts, ranging from a median of 34.5 months in arm H to approximately 58–66.5 months in arms D–F [20]. Furthermore, more than half of the patients in each innovaTV 205 cohort received at least one subsequent systemic therapy, which may have influenced OS [20].

Accordingly, the median OS values of 25.5–30.7 months reported in the first-line innovaTV 205 cohorts cannot be directly compared with the median OS of approximately 11.5–12.1 months reported in later-line monotherapy studies. These differences may reflect treatment line, regimen composition, patient selection, subsequent therapy, and follow-up duration rather than the effect of TV alone.

Overall, variability in efficacy outcomes across the innovaTV programmes appears to reflect differences in treatment line, previous therapy, regimen composition, study design, population selection, sample size, outcome assessment, subsequent treatment, and follow-up duration rather than inconsistent activity of TV. The monotherapy studies provide relatively consistent evidence of single-agent activity in previously treated disease. The higher response rates observed with first-line combinations in innovaTV 205 are promising but should remain considered exploratory until confirmed in adequately powered randomised trials.

### 4.2. Clinical Relevance of Patient-Reported Outcome

In recurrent or metastatic cervical cancer, the clinical value of treatment cannot be assessed solely on the basis of radiological response or survival. Patients with recurrent or metastatic cervical cancer may experience symptoms associated with progressive disease, previous treatment, and treatment-related toxicity. Preservation of daily functioning and health-related quality of life (QoL) is therefore particularly important in the second- and third-line settings, where treatment is predominantly palliative [4,21].

The patient-reported outcomes from innovaTV 301 complement the demonstrated improvements in OS, PFS, and ORR [19]. Despite the characteristic AEs profile of TV, global health status and quality of life remained stable from baseline to cycle 5 [19]. The estimated deterioration in the EORTC QLQ-C30 global health status/QoL score was smaller with TV than with chemotherapy, and a greater proportion of patients receiving TV achieved a clinically meaningful improvement of at least 10 points [19]. These findings suggest that the survival benefit of TV was accompanied by preservation of patients’ perceived overall health rather than by a clinically relevant worsening of QoL.

This observation is clinically meaningful because TV is associated with AESIs, particularly ocular events, peripheral neuropathy, and bleeding [15,16,17,18,19,20]. Although such events may negatively affect physical functioning and treatment experience, most can be mitigated through structured ophthalmic prophylaxis, close neurological monitoring, dose interruption, and dose modification [15,16,17,18,19,20,21,22,23,24,25,26,27,28]. Effective toxicity management may therefore be important not only for maintaining treatment exposure but also for preserving QoL.

Nevertheless, the PRO findings require cautious interpretation. The cycle 5 analysis included patients who were alive and remained on treatment, which may introduce attrition or survivor bias by underrepresenting patients with rapid progression, severe toxicity, or early treatment discontinuation [19]. Moreover, innovaTV 301 was an open-label study, and awareness of treatment allocation may have influenced self-reported outcomes. The available publication primarily reported changes in the EORTC QLQ-C30 global health status/QoL score, while detailed longitudinal results from the cervical cancer-specific QLQ-CX24 and EQ-5D-5L instruments were limited [19]. Consequently, the available evidence supports short-term preservation of overall QoL, but does not yet provide a comprehensive assessment of symptom-specific, sexual, emotional, and long-term functional outcomes.

Future studies should report PRO completion rates at all assessment points, time to definitive deterioration, symptom-specific trajectories, and outcomes after treatment discontinuation. Such analyses will be particularly important for determining whether the higher response rates achieved with intensive TV-based combinations translate into meaningful patient-centred benefits, given the increased incidence of grade ≥ 3 adverse events and treatment discontinuation observed with multi-agent regimens [20].

### 4.3. Summary and Synthesis of the Safety Profile of Tisotumab Vedotin (TV)

Analysis of the data presented from the innovaTV 301, innovaTV 204, innovaTV 201 and innovaTV 205 clinical trials clearly indicates that TV is characterised by a predictable and reproducible toxicity profile [15,16,17,18,19,20]. Although the incidence of (TEAEs) of any severity in the study groups reached 100%, the majority of these were Grade 1 or 2 complications on the CTCAE scale [18,19]. A comparison of studies using TV as monotherapy and in multi-agent regimens also allows for a comprehensive assessment of the toxicity profile. Intensification of treatment by adding other drugs resulted in a significant increase in the incidence of Grade ≥3 TEAEs (an increase from 28–43% in monotherapy to 86.6% in the four-drug regimen), which also translates into a higher rate of treatment discontinuation (12–13% vs. 24.2–55.3%) [15,16,17,18,19,20].

The mechanism of action of the drug is of key importance for understanding the occurrence of AESIs, i.e., ophthalmic, neurological and haemorrhagic complications, in the context of TV. The targeting of the antibody against TF upon binding releases cytotoxic MMAE not only in the tumour microenvironment but also in sites where TF is physiologically present, namely in corneal and conjunctival epithelial cells, in the stromal cells surrounding blood vessels, and in the outer layers of blood vessels. TF also plays a key role in the coagulation cascade; therefore, its inhibition leads to local haemostatic disorders, manifesting as nosebleeds, although this can sometimes result in severe bleeding from the genital tract or the urinary system. Peripheral neuropathy is also a result of systemic exposure to MMAE, which exhibits an affinity for neurons by blocking microtubule polymerisation [25].

### 4.4. Practical Management of Toxicity

The implementation of prophylactic regimens and modifications to treatment in response to adverse effects is crucial for maintaining treatment continuity and minimising the risk of severe, irreversible treatment-related complications [19]. Official registration guidelines from the U.S. Food and Drug Administration (FDA) and the European Medicines Agency (EMA) define precise management protocols for adverse events, particularly in the case of AESIs and severe skin reactions [26,27].

Ocular complications are the most common manifestation of TV toxicity, the most frequent of which are conjunctivitis, dry eye, and corneal inflammation or ulceration [15,18]. The results of the innovaTV 201 study showed that the implementation of preventive strategies is necessary, reducing the incidence of conjunctivitis by nearly half (56% vs. 29%) [16]. The measures recommended by the FDA/EMA include a full ophthalmological examination prior to the start of therapy and before each subsequent cycle, the administration of vasoconstrictor eye drops immediately before the start of the TV infusion to reduce conjunctival vascularisation and limit drug distribution, as well as the topical application of corticosteroids to reduce inflammation, and the use of cooling eye compresses during the infusion, also aimed at limiting blood flow in the eye tissues. There is also a ban on wearing contact lenses throughout the duration of TV therapy [25,26,27].

In the case of peripheral neuropathy, regular neurological assessment is required prior to each treatment cycle due to the cumulative nature of MMAE neurotoxicity [16,18]. The FDA/EMA regulatory agencies have strictly defined dosage adjustment thresholds depending on the severity of symptoms. At grade 1, treatment is continued at the standard dose (2.0 mg/kg) under close monitoring. In the event of grade 2 neuropathy, administration of the next dose must be suspended until clinical improvement is observed, and the drug is reintroduced at a reduced dose of 1.3 mg/kg; in the event of recurrence, the dose is reduced to 0.9 mg/kg. The occurrence of this complication at grade 3 or 4 is an absolute indication for immediate discontinuation of TV therapy [26,27].

Management of bleeding events varies depending on the grade of the complications. In the case of Grade 1 major bleeding, the main approach is observation and, if necessary, symptomatic treatment. Grade 2 bleeding in sites other than the CNS or lungs requires discontinuation of the drug until haemoglobin levels stabilise and the bleeding has ceased. Resumption of therapy begins with a full or reduced dose (1.3 mg/kg). However, any grade 3 bleeding on a second recurrence and grade 4 bleeding, or any haemorrhagic events in the CNS, absolutely require discontinuation of TV treatment [16,25,26,27].

Although SJS is rare and has only been reported in the innovaTV 301 trial, it is potentially fatal [19]. Current FDA warnings and EMA guidelines mandate immediate discontinuation of the infusion or suspension of the next cycle of TV at the slightest clinical suspicion of SCARs. The patient must then be referred for urgent dermatological consultation, a skin biopsy must be performed, and intensive systemic treatment must be initiated. Confirmation of a diagnosis of grade ≥3 SJS necessitates a lifelong ban on the use of tisotumab vedotin [25,26,27].

**Figure 4:**
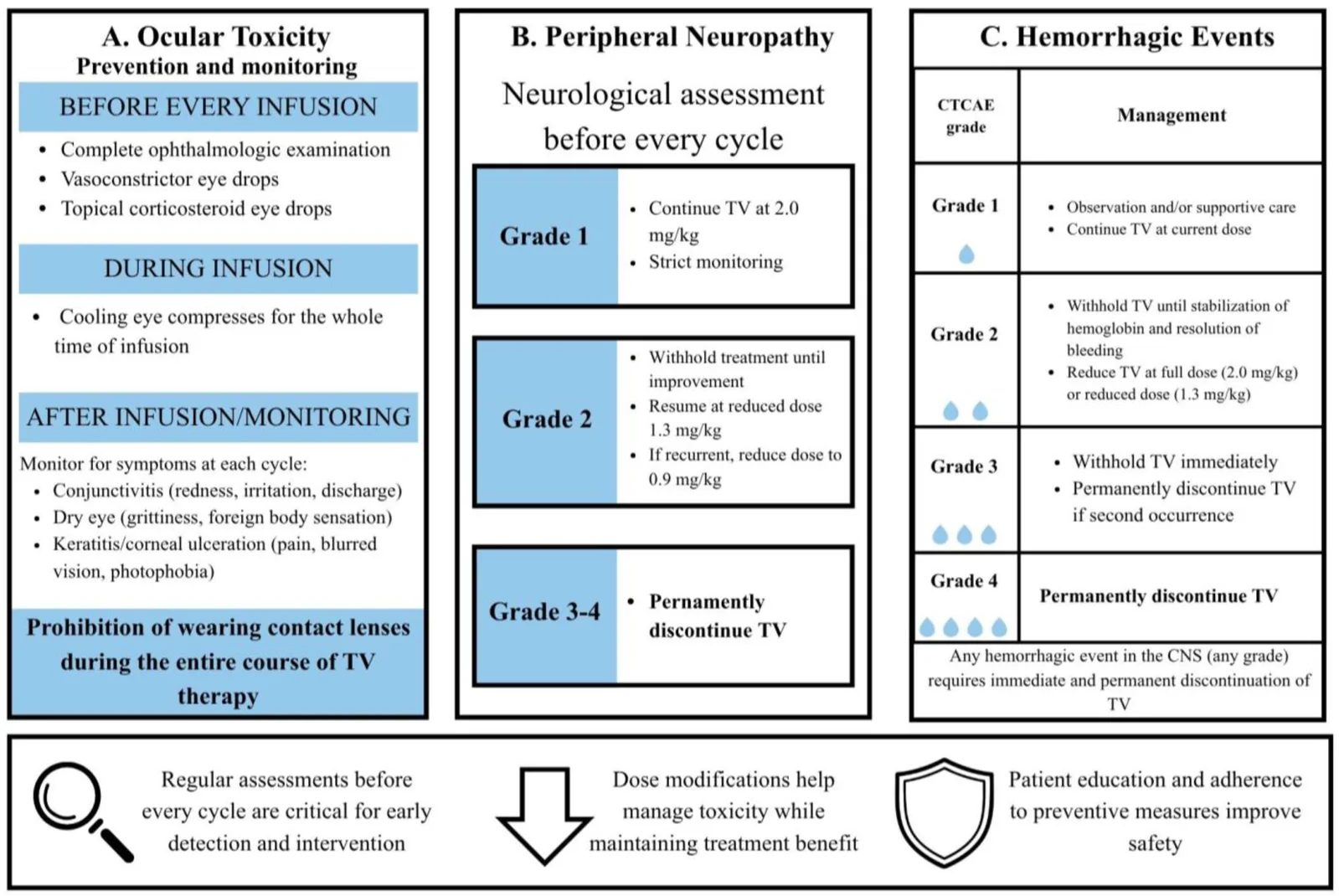
Management of key toxicities associated with tisotumab vedotin (TV).

Additionally, due to the risk of infusion-related reactions (IRRs), which in early-phase trials affected up to 12% of patients, manifesting mainly as acute abdominal pain and vomiting, medical supervision is recommended during infusion and, in the event of mild IRRs-the infusion rate should be slowed [18]. Severe or recurrent reactions (grade ≥ 3) require immediate discontinuation of the drug, administration of antihistamines and glucocorticosteroids, and exclude the patient from further TV therapy [26,27]. The management of key toxicities associated with TV is shown in Figure 4.

### 4.5. Future Research Directions

Despite the proven efficacy of TV, research in this area continues to evolve. A recent analysis of the international clinical trials registry ClinicalTrials.gov has shown that current studies are focused on optimising TV treatment strategies to improve efficacy and reduce adverse effects. There are 10 clinical trials involving TV, all of which are interventional; of these, 6 have already been completed (NCT03438396, NCT03913741, NCT03245736, NCT04697628, NCT02552121, NCT02001623), 1 has been terminated (NCT03786081), 2 are in the active phase with recruitment completed (NCT06459180), and 1 is in the recruitment phase (NCT06952660). Four trials are Phase I/II trials, three are Phase II trials, two are Phase III trials, and one is a Phase IV trial. A detailed description is provided in Table 4.

An important area of research involves the search for new molecular compounds for TV within the context of multi-drug therapies. Clinical trials are currently underway to assess the safety and efficacy of combining TV with new immunocompatible molecules and small-molecule angiogenesis inhibitors other than bevacizumab [28,29]. These studies aim to determine whether combination therapies can reduce the incidence of severe peripheral neuropathies and ophthalmic complications, which currently constitute the main cause of treatment discontinuation in intensive regimens [29,30]. Concurrently, future analyses must focus on identifying predictive biomarkers of tissue response [31]. Although TV targets TF, clinical analyses to date have not demonstrated a linear correlation between the degree of TF overexpression in immunohistochemistry (IHC) and the degree of clinical benefit. Translational research in upcoming clinical trials is focusing on the assessment of tumour heterogeneity, the dynamics of circulating tumour DNA (ctDNA) and the soluble form of TF (sTF) in plasma as potential tools for personalised therapy and the early detection of resistance to MMAE [23,31].

### 4.6. Limitations of the Review

Despite the rigorous and systematic methodology applied to the scientific literature on the efficacy of TV in the treatment of cervical cancer, this review has certain methodological and substantive limitations that should be taken into account when interpreting the results. Another limitation is that a quantitative meta-analysis was not performed because of substantial clinical and methodological heterogeneity among the included studies. Differences in treatment setting, combination regimens, endpoints, and follow-up duration precluded formal statistical pooling of efficacy outcomes. The conclusions are based only on qualitative evidence synthesis.

#### 4.6.1. Differences in Data Maturity and Study Design

The main limitation is the inclusion of studies with different study designs and varying degrees of clinical maturity. InnovaTV 201 and innovaTV204 are early-phase, single-arm, open-label studies without a control group. The absence of a direct control arm at this stage limits the ability to draw direct conclusions regarding the superiority of TV over other treatment methods [16,17,18].

In contrast, although the InnovaTV205 trial provides encouraging efficiency results regarding OS and DOR for combination regimens, it was characterised by a relatively small sample size [20]. Such small patient cohorts increase the risk of statistical error and require confirmation in multicentre, randomised phase III trials.

#### 4.6.2. Reporting of Efficacy Parameters According to Different Criteria

In some of the analysed studies, such as innovaTV 201, parameters such as ORR or PFS were reported twice: according to the assessment of the physicians and that of the Independent Review Committee (IRC) [10,11]. Differences in these readings, amounting to several percentage points, introduce a degree of heterogeneity into the data [32]. This makes it difficult to perform an unambiguous, linear comparison of results between cohorts without the risk of error.

#### 4.6.3. Limited Breadth of the Available Evidence

This review included six publications, which relate solely to four main clinical trial programmes (innovaTV 201, 204, 205 and 301) [15,16,17,18,19,20]. Several publications presented secondary analyses, long-term follow-up, subgroup analyses or patient-reported outcomes derived from the same study populatins, rather than from independent clinical trials. Consequently, the available data are derived from a relatively small number of distinct patient cohorts, which limits the scope and independence of the evidence base. The results should be interpreted as reflecting the current stage of clinical development of TV, rather than as definitive evidence applicable to all clinical situations. Further independent randomised trials and studies are required to confirm these results in broader populations.

#### 4.6.4. Impact of Risk of Bias on the Interpretation of the Outcomes

The efficacy findings presented in this review should be interpreted in light of the methodological limitations of the included studies. Although the phase III randomised innovaTV 301 trial demonstrated a low overall risk of bias, most of the available evidence comes from early-phase, single-arm, open-label studies without a control group [19]. Such studies are more prone to selection bias, confounding factors and overestimation of treatment effects, particularly in the case of endpoints based on treatment response. The absence of randomisation and direct comparison groups limits the strength of causal inferences regarding treatment efficacy. Differences in patient populations, treatment regimens, combination therapies, sample sizes and duration of follow-up make it impossible to draw reliable comparisons of ORR, PFS or OS rates between individual trials. Consequently, the promising efficacy observed in early-phase trials should be regarded as a starting point for formulating hypotheses and interpreted in conjunction with more robust evidence from a randomised phase III trial. While the consistency of the efficacy results across the various studies reinforces confidence in the clinical efficacy of TV, further randomised trials are needed to define its optimal role in different therapeutic settings more precisely.

## 5. Conclusions

TV is a drug for patients with recurrent or metastatic CC. It is the first ADC to target TF. It induces cancer cell death regardless of the presence of specific immune receptors on the cells. For this reason, TV is also an effective treatment option for PD-1- negative patients.

Numerous clinical trials have demonstrated the efficiency of TV and opened up new avenues for the use of TV as a first-line treatment—particularly in combination with immunotherapy and platinum-based agents. Unfortunately, they have also shown that, due to its affinity for TF, it is associated with a high incidence of adverse effects. However, when TV is used as monotherapy, adverse effects are generally manageable. The use of TV in combination with other drugs during intensified treatment leads to increased toxicity, particularly within the neurological and haematopoetic systems. Serious complications may lead to interruption or even premature termination of treatment. For this reason, an multidisciplinary approach to the patient should be adopted, with particular attention to the prevention and early management of ocular complications, peripheral neuropathy and haemorrhagic events. Adherence to safety protocols helps to reduce the incidence of adverse effects—in some cases by as much as half.

These conclusions are based primarily on four closely related clinical trials, with several included publications representing secondary analyses of the same patient cohorts. Therefore, although the evidence is consistent and supported by randomised phase III data, the findings should not be overgeneralized. Further independent randomised trials and longer follow-up studies are needed to better define the optimal positioning of TV, identify patients most likely to benefit, and confirm the durability and generalizability of its clinical benefits.

## Figures and Tables

**Table 1:** Characteristics and quantitative synthesis of the clinical efficacy outcomes of TV in the studies included in the review [15,16,17,18,19,20].

STUDY	Objective Response Rate (ORR)	Disease Control Rate (DCR)	Mediana PFS (msc)	Mediana OS (msc)	Czas Trwania Odpowiedzi (DOR–*Duration of Response*) (msc)
**innovaTV 301(phase 3)** **(N = 253)** Recurrent/metastatic cervical cancer after prior treatment vs. chemotherapy	17.8%	75.9%	4.2 vs. 2.9	11.5 vs. 9.5	5.3
**innovaTV 204 (phase 2)** **(N = 101)**	24%	72%	4.2	12.1	8.3 Durable response had not yet been reached in all patients at the time of analysis.
**innovaTV 201** (phase 1/2) (N = 55)	24% (IRC assessmen = 22%)	Not reported	4.2	Early phase of the study	4.2–6 (some patients maintained responses for >8 months)
innovaTV 205 (phase Ib/II; N = 139 treated patients; n = 137 efficacy-evaluable patients) **Arm D: TV + Carboplatin (1L)** **(N = 33)** **Arm E: TV + Pembrolizumab (1L)** **(efficacy population, N = 32)** **Arm F: TV + Pembrolizumab (2L/3L)** **(efficacy population, N = 34)** **Arm H:TV + carboplatin + pembrolizumab ± bevacizumab (1L)** **(N = 38)**	35.3–54.5% 54.5% 40.6% 35.3% 65.8%	73.5–98.1%	5.3–6.9 6.9 5.3 5.6	15.3–30.7 25.4 30.7 15.3 28.0	8.6–18.2 (arm E- NR) 8.6 NR 14.1–18.2 13.3

OS—overall survival, ORR—objective response rate, 95% CI—95% confidence interval, DCR—disease control rate, DOR—duration of response (duration of response), HR—hazard ratio, 1L = first-line treatment, 2L = second-line treatment, 3L = third-line treatment, IRC = Independent Review Committee, NR—Not Reached.

**Table 2:** Summary of the safety and toxicity profile of tisotumab vedotin (TV) in the innovaTV 301, innovaTV 204, innovaTV 201 and innovaTV 205 clinical trials [15,16,17,18,19,20].

Parameter/Study	innovaTV 301 (n = 101) Phase III	innovaTV 204 (n = 101) Phase II	innovaTV 201 (n = 147, of Whom n = 34 Had Cervical Cancer/n = 55 Had Cervical Cancer) Phase I/II	innovaTV 205 (n = 139) Phase Ib/II
Median exposure (time)	3.5 months	4.2 months	No data available/No data available	from 34.5 to 66.5 months
TEAE (any degree)	100%	92%	100%/100%	100%
TEAE grade ≥ 3	42.9%	28.0%	56,0% (in cervical cancer), 42.0% (in the entire study group)/56%	D: 72.7% E: 45.5% F: 48.6% H: 86.8%
SAE	14.3%	13.0%	46.0% (in the cervical cancer group)	In accordance with the profiles of the individual medicines
Drug-related deaths (Grade 5.)	0%	1.0%(n = 1; anafilactic shock)	0.7%/0%	E: 3.0% (n = 1; hypowolemic shock)
Dose adjustment (Reduction/Break)	44.9%	22%/24%	18%/13%	Frequently administered via the H arm (due to haematologicaltoxicity)
Complete discontinuation of treatment	12.2%	12.0–13.0%	22.0% (cervical cancer cohort) 9.0% (total population)/18%	D/E: 24.2% F: 34.3% H: 55.3%

**Table 3:** Toxicity profile of particular clinical relevance for tisotumab vedotin (TV) in the innovaTV 301, innovaTV 204, innovaTV 201 and innovaTV 205 clinical trials [15,16,17,18,19,20].

Parameter/Study	innovaTV 301 (n = 101) Phase III	innovaTV 204 (n = 101) Phase II	innovaTV 201 (n = 147, of Whom n = 34 Had Cervical Cancer/n = 55 Had Cervical Cancer) Phase I/II	innovaTV 205 (n = 139) Phase Ib/II
Ocular toxicity	53.1%	53.0%	60.0%/60% following the implementation of risk-mitigation measures	D: 66.7% E: 69.7% F:54.3% H: 78.9% (No grade 4.)
Peripheralneuropathy	38.8%	42.0%	43.0%/55%	D: 60.6% E: 54.5% F: 40.0% H: 65.8% (No grade 4.)
Bleeding episodes	42.9%	51.0%	69.0%/73%	D: 57.6% E: 66.7% F: 68.6% H: 60.5% (Grade 4 is present in F [2.9%] and H [5.3%])

**Table 4:** Clinical trials landscape of tisotumab vedotin in solid tumours and cervical cancer: study characteristics, phases, and primary outcomes.

	NCT Number	Study Status	Phase	Interventions	Conditions	Primary Outcomes
1.	NCT03438396	COMPLETED	PHASE2	TV	Cervical cancer	ORR
2.	NCT03913741	COMPLETED	PHASE1|PHASE2	TV	PART 1 ONLY: Solid tumours PART 2 ONLY: Cervical cancer	AEs, SAEs, DLTs, MTD/RP2D, Cmax, AUC, CL, T1/2, ADA
3.	NCT03245736	COMPLETED	PHASE2	TV	Ovary Cancer, Cervix Cancer, Endometrium Cancer, Bladder Cancer, Prostate Cancer, Oesophagus Cancer, Lung Cancer, Nonsmall Cell Squamous Cell, Carcinoma of the Head and Neck	TEAEs
4.	NCT03786081	TERMINATED (Decided by sponsor based on strategic decisions)	PHASE1|PHASE2	TV/Bevacizumab/Pembrolizumab/Carboplatin	Cervical Cancer	DLTs, MTD, RP2D, ORR
5.	NCT04697628	COMPLETED	PHASE3	TV	Cervical Cancer	OS
6.	NCT02552121	COMPLETED	PHASE1|PHASE2	TV(HuMax-TF-ADC)	Bladder Cancer, Cervix Cancer, Endometrium Cancer, Oesophagus Cancer, Lung Cancer (NSCLC), Ovary Cancer, Prostate Cancer (CRPC)	Safety and toxicity profile: AEs, SAEs, IRRs
7.	NCT02001623	COMPLETED	PHASE1|PHASE2	TV (HuMax-TF-ADC)	Bladder Cancer, Cervix Cancer, Endometrium Cancer, Oesophagus Cancer, Lung Cancer(NSCLC), Ovary Cancer, Prostate Cancer (CRPC), Squamous Cell Carcinoma of the Head and Neck (SCCHN)	TEAEs
8.	NCT03485209	ACTIVE/NOT RECRUITING	PHASE2	TV/Pembrolizumab/Carboplatin/Cisplatin	Non-Small-Cell Lung, Squamous Cell of Head and Neck, Colorectal Neoplasms, Exocrine Pancreatic Cancer	ORR
9.	NCT06459180	ACTIVE/NOT RECRUITING	PHASE3	TV/Sacituzumab Tirumotecan vs. TPC (Pemetrexed/Topotecan/Vinorelbine/Gemcitabine/Irinotecan)	Cervical Cancer	Run-in phase: ORR, AEs, Phase III: OS
10.	NCT06952660	RECRUITING	PHASE4	TV	Cervical Cancer	Safety and toxicity profile connected with ocular adverse events

AEs—Adverse Events; AUC—Area Under the Plasma Concentration–Time Curve; CL—Systemic Clearance; Cmax—Maximum Observed Plasma Concentration; DLTs—Dose-Limiting Toxicities; IRRs—Infusion-Related Reactions; MTD—Maximum Tolerated Dose; NCT—National Clinical Trial number; ORR—Objective Response Rate; OS—Overall Survival; RP2D—Recommended Phase 2 Dose; SAEs—Serious Adverse Events; T1/2—Elimination Half-Life; TEAEs—Treatment-Emergent Adverse Events; TPC—Treatment of Physician’s Choice; TV—Tisotumab Vedotin.

## Data Availability

No new data were created or analysed in this study. Data sharing is not applicable to this article as it is a systematic review based on previously published research. The data presented in this study are available within the article (including tables and figures) and the cited references.

## Supplementary Materials

The following supporting information can be downloaded at: https://www.mdpi.com/article/10.3390/cancers18162685/s1, File S1. PRISMA 2020 Checklist.

## Abbreviations

The following abbreviations are used in this manuscript:

1L	First-line treatment
2L	Second-line treatment
3L	Third-line treatment
ADC	Antibody–drug conjugates
AEs	Adverse Events
AESIs	Adverse events of special clinical significance
AUC	Area Under the Plasma Concentration–Time Curve
CC	Cervical cancer
Cmax	Maximum Observed Plasma Concentration
CR	Complete response
CT	Computed tomography
CTCAE	Common Terminology Criteria for Adverse Events
CTRT	Concurrent chemoradiotherapy
CL	Systemic Clearance;
DCR	Disease control rate
DLTs	Dose-Limiting Toxicities
DOR	Duration of response
ESGO	European Society of Gynaecological Oncology
Fc	Crystallised fragment of an antibody
FcRn	Neonatal Fc receptor
FST	Fertility-sparing treatment
HR	Hazard ratio
IGABT	Image-guided adaptive brachytherapy
IRC	Independent Review Committee
IRRs	Infusion-Related Reactions
LACC	Locally advanced cervical cancer
mAb	Monoclonal antibody
MTD	Maximum Tolerated Dose
MMAE	Monomethylauristatin E
MRI	Magnetic resonance imaging
NCT	National Clinical Trial number
NR	Not reached
ORR	Objective response rate
OS	Overall survival
PALND	Bilateral pelvic lymphadenectomy
PET-CT	Positron emission tomography–computed tomography
PFS	Progression-free survival
PICO	Population, Intervention, Comparison, and Outcome
PR	Partial response
PRISMA	Preferred Reporting Items for Systematic Reviews and Meta-Analyses
PRO	Patient-reported outcome
PROSPERO	International Prospective Register of Systematic Reviews
QoL	Ouality of Life
RECIST	Response Evaluation Criteria in Solid Tumours
RoB 2.0	Cochrane Risk of Bias tool for randomised trials
RP2D	Recommended Phase 2 Dose
SAEs	Serious adverse events
SJS	Stevens–Johnson syndrome
T1/2	Elimination Half-Life
TEAEs	Treatment-emergent adverse events
TF	Tissue factor
TPC	Treatment of Physician’s Choice
TV	Tisotumab vedotin
95% CI	95% confidence interval

## References

[1] Bray F., Laversanne M., Sung H., Ferlay J., Siegel R.L., Soerjomataram I., Jemal A. (2024). Global cancer statistics 2022: GLOBOCAN estimates of incidence and mortality worldwide for 36 cancers in 185 countries. CA Cancer J. Clin..

[2] Jouya S., Shahabinia Z., Mazidimoradi A., Allahqoli L., Salehiniya H., Lee D.-Y. (2026). Cervical Cancer Epidemiology: Global Incidence, Mortality, Survival, Risk Factors, and Equity in HPV Screening and Vaccination. J. Clin. Med..

[3] Pötter R., Tanderup K., Schmid M.P., Jürgenliemk-Schulz I., Haie-Meder C., Fokdal L.U., Sturdza A.E., Hoskin P., Mahantshetty U., Segedin B. (2021). MRI-guided adaptive brachytherapy in locally advanced cervical cancer (EMBRACE-I): A multicentre prospective cohort study. Lancet Oncol..

[4] Cibula D., Raspollini M.R., Planchamp F., Centeno C., Chargari C., Felix A., Fischerová D., Jahnn-Kuch D., Joly F., Kohler C. (2023). ESGO/ESTRO/ESP Guidelines for the management of patients with cervical cancer—Update 2023*. Int. J. Gynecol. Cancer.

[5] Liontos M., Kyriazoglou A., Dimitriadis I., Dimopoulos M.-A., Bamias A. (2019). Systemic therapy in cervical cancer: 30 years in review. Crit. Rev. Oncol..

[6] Galicia-Carmona T., Arango-Bravo E.A., Coronel-Martínez J.A., Cetina-Pérez L., Vanoye-Carlo E.G., Villalobos-Valencia R., García-Pacheco J.A., Cortés-Esteban P. (2024). Advanced, recurrent, and persistent cervical cancer management: In the era of immunotherapy. Front. Oncol..

[7] Wei H., Zhang Y., Lu Y., Zou Y., Zhou L., Qin X., Jiang Q. (2025). Is ADC a rising star in solid tumor? An umbrella review of systematic reviews and meta-analyses. BMC Cancer.

[8] Fu Z., Li S., Han S., Shi C., Zhang Y. (2022). Antibody drug conjugate: The “biological missile” for targeted cancer therapy. Signal Transduct. Target. Ther..

[9] Wang R., Hu B., Pan Z., Mo C., Zhao X., Liu G., Hou P., Cui Q., Xu Z., Wang W. (2025). Antibody–Drug Conjugates (ADCs): Current and future biopharmaceuticals. J. Hematol. Oncol..

[10] U.S. Food and Drug Administration Drugs@FDA: FDA-Approved Drugs—TIVDAK (tisotumab vedotin-tftv), BLA 761208, Original Approval Letter, 20 September 2021. https://www.accessdata.fda.gov/drugsatfda_docs/appletter/2021/761208Orig1s000_Corrected_ltr.pdf.

[11] U.S. Food and Drug Administration Drugs@FDA: FDA-Approved Drugs—TIVDAK (tisotumab vedotin-tftv), BLA 761208, Supplement Approval Letter (SUPPL-7), 29 April 2024. https://www.accessdata.fda.gov/drugsatfda_docs/appletter/2024/761208Orig1s007ltr.pdf.

[12] European Medicines Agency Tivdak (tisotumab vedotin)—European Public Assessment Report (EPAR) Overview, EMA/54932/2025. Authorised 28 March 2025. https://www.ema.europa.eu/en/medicines/human/EPAR/tivdak.

[13] Zeng S., Li X., Xiao S., Yang P., Lin C., Chen H., Zhao H., Zhan J., Xiao X. (2025). The efficacy and safety of Tisotumab vedotin in the treatment of recurrent/metastatic cervical cancer: A systematic review and meta-analysis of single-arm studies. Front. Pharmacol..

[14] Higgins J.P.T., Thomas J., Chandler J., Cumpston M., Li T., Page M.J., Welch V.A. (2019). Cochrane Handbook for Systematic Reviews of Interventions.

[15] Yonemori K., Kuboki Y., Hasegawa K., Iwata T., Kato H., Takehara K., Hirashima Y., Kato H., Passey C., Buchbjerg J.K. (2022). Tisotumab vedotin in Japanese patients with recurrent/metastatic cervical cancer: Results from the innovaTV 206 study. Cancer Sci..

[16] de Bono J.S., Concin N., Hong D.S., Thistlethwaite F.C., Machiels J.-P., Arkenau H.-T., Plummer R., Jones R.H., Nielsen D., Windfeld K. (2019). Tisotumab vedotin in patients with advanced or metastatic solid tumours (InnovaTV 201): A first-in-human, multicentre, phase 1–2 trial. Lancet Oncol..

[17] Hong D.S., Concin N., Vergote I., de Bono J.S., Slomovitz B.M., Drew Y., Arkenau H.-T., Machiels J.-P., Spicer J.F., Jones R. (2020). Tisotumab Vedotin in Previously Treated Recurrent or Metastatic Cervical Cancer. Clin. Cancer Res..

[18] Coleman R.L., Lorusso D., Gennigens C., González-Martín A., Randall L., Cibula D., Lund B., Woelber L., Pignata S., Forget F. (2021). Efficacy and safety of tisotumab vedotin in previously treated recurrent or metastatic cervical cancer (innovaTV 204/GOG-3023/ENGOT-cx6): A multicentre, open-label, single-arm, phase 2 study. Lancet Oncol..

[19] Yonemori K., Nishio S., Suzuki S., Hasegawa K., Yunokawa M., Yamaguchi S., Okamoto A., Nakamura K., Kamiura S., Fujiwara K. (2025). Tisotumab vedotin in Japanese patients with recurrent or metastatic cervical cancer: Results from the innovaTV 301/ENGOT-cx12/GOG-3057 trial. Int. J. Gynecol. Cancer.

[20] Van Nieuwenhuysen E., Vergote I., Randall L.M., Tromp J., Lorusso D., O’CEarbhaill R.E., Boere I., Pisano C., Sanchez L.M., Banerjee S. (2026). Tisotumab vedotin plus carboplatin or pembrolizumab in recurrent or metastatic cervical cancer: 5-year results from the innovaTV 205/ENGOT-cx8/GOG-3024 study. Gynecol. Oncol..

[21] Abu-Rustum N.R., Campos S.M., Amarnath S., Arend R., Barber E., Bradley K., Brooks R., Chino J., Chon H.S., Crispens M.A. (2025). Cervical Cancer, Version 2.2026, NCCN Clinical Practice Guidelines In Oncology. J. Natl. Compr. Cancer Netw..

[22] Chung H., Ros W., Delord J.-P., Perets R., Italiano A., Shapira-Frommer R., Manzuk L., Piha-Paul S., Xu L., Zeigenfuss S. (2019). Efficacy and Safety of Pembrolizumab in Previously Treated Advanced Cervical Cancer: Results From the Phase II KEYNOTE-158 Study. J. Clin. Oncol..

[23] Luu K., Chu A., Chang B. (2022). A review of the novel tissue factor antibody–drug conjugate: Tisotumab vedotin. J. Oncol. Pharm. Pract..

[24] Colombo N., Dubot C., Lorusso D., Caceres M.V., Hasegawa K., Shapira-Frommer R., Tewari K.S., Salman P., Usta E.H., Yañez E. (2021). Pembrolizumab for Persistent, Recurrent, or Metastatic Cervical Cancer. N. Engl. J. Med..

[25] Arn A.-C.C.R., Halla M.K.J., Gill R.S. (2023). Tisotumab Vedotin Safety and Tolerability in Clinical Practice: Managing Adverse Events. J. Adv. Pract. Oncol..

[26] U.S. Food and Drug Administration (FDA) TIVDAK (tisotumab vedotin-tftv) Injection, for Intravenous Use: Full Prescribing Information [Revised 2024]. https://www.accessdata.fda.gov/drugsatfda_docs/label/2024/761208s007lbl.pdf.

[27] European Medicines Agency (EMA) Tivdak (tisotumabum vedotinum): Aneks I—Charakterystyka Produktu Leczniczego [aktualizacja 2025/2026]. https://www.ema.europa.eu/pl/documents/product-information/tivdak-epar-product-information_pl.pdf.

[28] Mokresh M.E., Alomari O., Varda A., Akdag G., Odabas H. (2024). Safety and efficacy of tisotumab vedotin with cervical cancers: A systematic review and meta-analysis. J. Obstet. Gynaecol. Res..

[29] Kim S.K., Ursell P., Coleman R.L., Monk B.J., Vergote I. (2022). Mitigation and management strategies for ocular events associated with tisotumab vedotin. Gynecol. Oncol..

[30] Zhou K., Liu X., Zhu H. (2025). Overcoming resistance to antibody-drug conjugates: From mechanistic insights to cutting-edge strategies. J. Hematol. Oncol..

[31] Takakura T., Shimizu T., Yamamoto N. (2024). Antibody-drug conjugates in solid tumors; new strategy for cancer therapy. Ultrasound Med. Biol..

[32] Page M.J., McKenzie J.E., Bossuyt P.M., Boutron I., Hoffmann T.C., Mulrow C.D., Shamseer L., Tetzlaff J.M., Akl E.A., Brennan S.E. (2021). The PRISMA 2020 statement: An updated guideline for reporting systematic reviews. BMJ.

